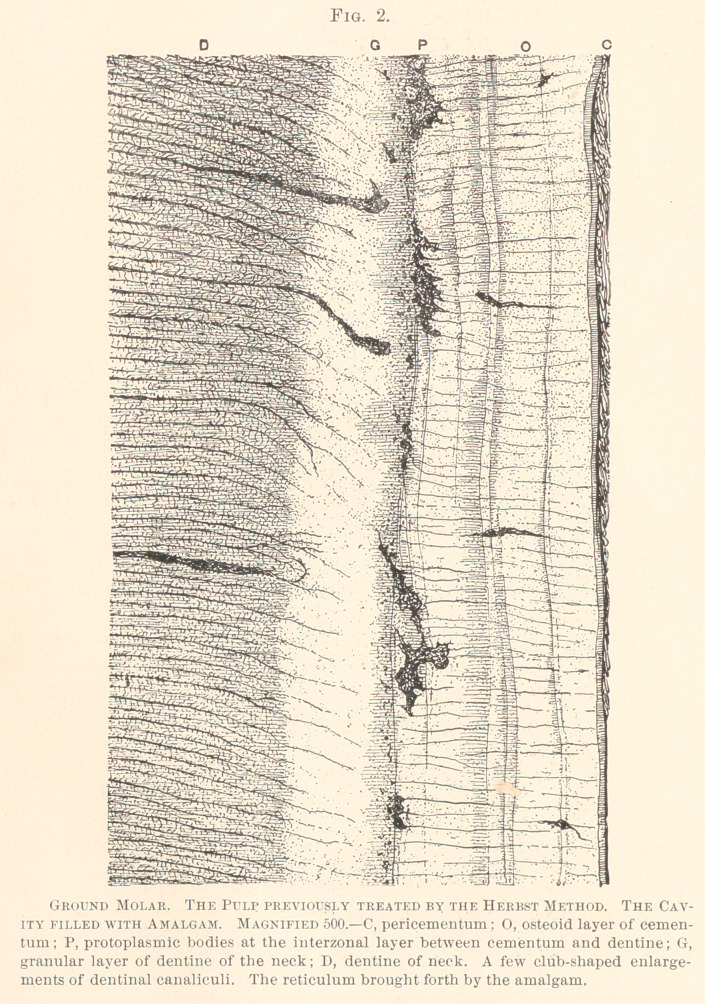# Demonstration of the Reticulum in Dentine with Low Powers of the Microscope

**Published:** 1892-09

**Authors:** C. Heitzmann


### International Dental Journal.

Vol. XIII. September, 1892. No. 9.

### Original Communications.[1]

### DEMONSTRATION OF THE RETICULUM IN DENTINE WITH LOW POWERS OF THE MICROSCOPE.[1]

BY C. HEITZMANN, M.D.

The first who maintained that the dentine has a reticular structure was C. F. W. Bödecker. In his paper, "The Distribution of Living Matter in Human Dentine, Cement, and Enamel" (*Dental Cosmos*, 1878–79), this writer says, "The basis-substance of the dentine shows a distinct, net-like structure. The light spaces surrounding the dentinal fibres send delicate elongations into the basis-substance, in which, through repeated branching, a light net-work is established, the meshes of which contain the decalcified (after treatment with solution of chromic acid), glue-yielding basis-substance. The finest offshoots of the dentinal fibres can be traced only into the mouths of the elongations of the canaliculi; on the periphery of the latter, owing to their great delicacy, the offshoots are lost to sight. Coarser offshoots of the dentinal fibres . . . traverse the basis-substance within its light net-work, at the same time uniting dentinal fibres directly, and sending slender conical offshoots into the light net-work of the basis-substance."

---

[1] The editor and publishers are not responsible for the views of authors of papers published in this department, nor for any claim to novelty, or otherwise, that may be made by them. No papers will be received for this department that have appeared in any other journal published in the country.

[1] Read at a meeting of the New York Odontological Society, May 17, 1892.

At the conclusion of his paper this author says, "The dentinal canaliculi are excavations in the basis-substance of the dentine, each containing in its centre a fibre of living matter. Besides the dentinal canaliculi, there exists an extremely delicate net-work within the basis-substance of the dentine, into which innumerable offshoots of the dentinal fibres pass. Although we cannot trace the living matter throughout the whole net-work in the basis-substance, we are justified in assuming that not only the dentinal canaliculi, but the whole basis-substance of the dentine, is also pierced by a delicate net-work of living matter."

From these quotations it is evident that Bödecker, with the method employed at that time,—*i.e.*, softening the dentine with solutions of chromic acid and slicing it with the razor,—saw a light reticulum, of which he assumed that it contained the living matter, judging from analogies in other varieties of tissue. The positive proof that Bödecker's assumption was correct was furnished much later, in 1887, by William Carr. He ground fresh dentine, kept moist with a weak solution of table-salt, to an extremely thin slab, which was left exposed to the influence of a half of one per cent. solution of chloride of gold for a number of hours, and was afterwards decalcified with a six-per-cent. solution of glacial acetic acid. The chloride of gold has been known for twenty-five years to stain a dark-violet color the living matter proper, having been first introduced into histology by Cohnheim for the purpose of staining the nerves of the cornea. Specimens obtained by William Carr beautifully demonstrated a dark-violet reticulum throughout the basis-substance of the dentine, being in direct union with the dentinal fibres as well as the coarser offshoots emanating therefrom. Some of these specimens were so convincing that I have demonstrated them with high powers of the microscope (one thousand to twelve hundred diameters, immersion) to a number of gentlemen of the medical and dental profession, in order to prove to them that all tissues, including the hardest of the teeth, were pervaded by living matter, the whole body thus being made a continuity, not only of protoplasm, but of its most essential constituent part, the contractile or living matter. Among those who publicly announced their conviction of this novel fact of such a great biological importance were Frank Abbott and W. H. Atkinson. Abbott speaks of this topic in the following words (Annual Address of the President of the American Dental Association, August 28, 1888): "The certainty of Bödecker's conclusions that the whole basis-substance of the dentine is pervaded by living matter has finally been reached by new methods adopted by William Carr, of New York, who, at his convenience, proposes to lay the results of his studies before the profession." William H. Atkinson, in his last paper, "The Origin of Pus" (*Journal of the American Medical Association*, 1889), expresses his conviction in the following way: "The presence of this reticulum was first established by C. F. W. Bödecker in 1878, who saw light rents in the basis-substance, and assumed them to hold living matter without being able to directly prove its presence. This proof has been recently furnished by William Carr, who, after decalcification of the dentine, rendered the reticulum visible by staining with a chloride of gold solution and osmic acid. These last results have not as yet been published by their observer."

Dr. William Carr, however, delayed finishing his work, and after waiting for years, I asked his permission to shift over the topic to another worker, John I. Hart, of New York. Dr. Carr kindly consented, and gave permission to Dr. Hart to use his specimens as best he could. Dr. Hart made a large number of new specimens, treated with chloride of gold, and published the results of his observations in a paper,—"Minute Structure of Dentine" (*Dental Cosmos*, September, 1891). So perfect were his specimens that he was enabled, by the configuration of the dentinal fibres and the reticulum, to tell at once whether the dentine examined came from a living or from a devitalized tooth. Besides, a large number of facts were obtained by him of the utmost interest and importance to the dental practitioner.

Still, the reticulum was demonstrable only with high powers of the microscope to eyes well trained for such observations, and thus it is explicable that some persons not in possession of trained eyes expressed their doubts as to the presence of such a reticulum.

Dr. Bödecker resumed work in my laboratory towards the end of 1891, with the purpose of studying the reaction in the dentine upon fillings of a widely different nature. He had collected a large number of teeth that were filled for months and years. The first tooth he ground thin was a bicuspid with two cavities in its crown; one filled with cement, the other with silver-amalgam,—both plugs having been in the cavities for years. How great was his surprise when, examining the border of the cavity previously filled with amalgam, he saw a dark-brown discoloration of the dentine, not directly along the border of the cavity, but some distance away from it, and in this brown zone the dentinal canaliculi, respectively their tenants, the dentinal fibres, crowded with black dots, and their offshoots rendered wonderfully plain by a black deposit. In the darkest portion the reticulum was easily seen with comparatively low powers of the microscope,—viz., five hundred diameters.

With the kind permission of Dr. Bödecker I lay before you this specimen (see Fig. 1) with a few brief remarks.

The border of the cavity is, in the upper portion, seen in the breadth of the specimen, and here it holds a number of black metallic particles, evidently not of the amalgam itself, but of a sulphur-combination of the silver or quicksilver. The zone of the dentine directly bordering the cavity is, apparently, destitute of structure, showing but faint traces of canaliculi, of a high refraction, obviously in a state of consolidation, a reactive process subsequent to the filling. Close beneath this consolidated layer follows a dark-brown zone, diffusively pigmented, and here we see the dentinal canaliculi crowded with black metallic particles, considerably widened and sending black offshoots into the basis-substance between the canaliculi. The offshoots are comparatively scarce and large towards the enamel, where the unstained canaliculi show only the usual bifurcation. The offshoots are more numerous upon entering the dark-brown region of the dentine, and in the darkest portion are so numerous and so delicate that the power of five hundred diameters is insufficient to dissolve the minutest branches, all of which inosculate into an extremely delicate black reticulum. All the features, the coarse and fine offshoots and their union into a reticulum, are identical with the image obtained by John I. Hart in living dentine, treated with chloride of gold.

How did the metallic particles reach the dentinal canaliculi, and produce such an image at a certain distance away from the border of the cavity? There is but one answer possible to this query. The metallic particles were taken into the living matter, probably before the consolidation was accomplished at the border of the cavity, and carried farther, at the same time rendering visible the reticulum, interconnecting the dentinal fibres. It would be impossible to understand the loading of the dentinal fibres, which run a parallel course and are separated from one another by the intervening basis-substance, unless by the presence of transverse connections of the fibres, as actually shown by the reticulum. This reticulum is, in fact, so plain that any tyro must see it, and the specimen alone is sufficient to remove the doubts of even the most sceptical minds.

A second specimen is placed under another microscope, likewise with a power of five hundred diameters. (See Fig. 2.)

The field exhibited is the neck [illegible] a molar whose pulp years ago was treated by Wilh. Herbst, of Bremen, Germany, with his method, recently made known in this country by Dr. Bödecker, at the meeting in Albany. Dr. Herbst applies cobalt upon the exposed or inflamed pulp of the crown; a few days afterwards he excises the pulp down to the root-canals; introduces tin-foil, which he grinds down at the bottom of the cavity, thus shutting off the pulp of the root-canals from contact with air, or other filling materials placed into the cavity of the crown. Two such molars were sent by Dr. Herbst to Dr. Bödecker, and the latter kindly consented to have these specimens exhibited at this meeting. The remarkable outcome of Bödecker's examination of the teeth sent by Herbst is that the pulp remains alive in the root-canal, and is sufficient to endow with life the whole tooth, though its crown is entirely deprived of its pulp. This fact becomes intelligible only upon the presence of interconnections between the parallel dentinal fibrillæ; for the dentinal fibres could not possibly be kept alive in the crown, unless by unions with the fibrillæ of the roots, the only ones kept directly alive by the pulp-tissue of the roots. This is an indirect, though stringent proof of the presence of the reticulum. The direct proof is furnished by the visible interconnections of the fibrillæ along the neck of the tooth, of a brownish color, in a slightly-brownish basis-substance. After having examined many hundreds of teeth under the microscope in all sorts of pathological changes, I have never met with such a discoloration in the dentine so frequently observed in the enamel. It is quite possible and reasonable to assume that the metallic salt, originated at the border of the cavity, was transferred into the dentinal fibres and their offshoots, and was, at last, deposited in the fibrillæ and their offshoots in the region of the neck, which, as John I. Hart's researches prove, abounds in living matter far in excess of any other portion of the dentine. This, I admit, is an hypothesis, to which I resort for lack of a better explanation; it is nearest to my mind, after thirty-two years of study of the teeth, having become so thoroughly convinced of the life of all constituent tissues of the teeth. The specimen under consideration exhibits an ill-calcified dentine, for it shows numerous protoplasmic bodies at the border of the dentine towards the osteoid layer of the cementum, and club-shaped enlargements of some dentinal fibrillæ. The granular layer of Tomes in the dentine of the neck destitute of dentinal canaliculi is narrower than in thoroughly calcified teeth; but present, nevertheless.

My intention in exhibiting these two specimens is to stop short all further doubts as to the reticular structure of the basis-substance of the dentine. In the face of the facts demonstrated I am entitled to expect such a result.

## Figures and Tables

**Fig. 1. f1:**
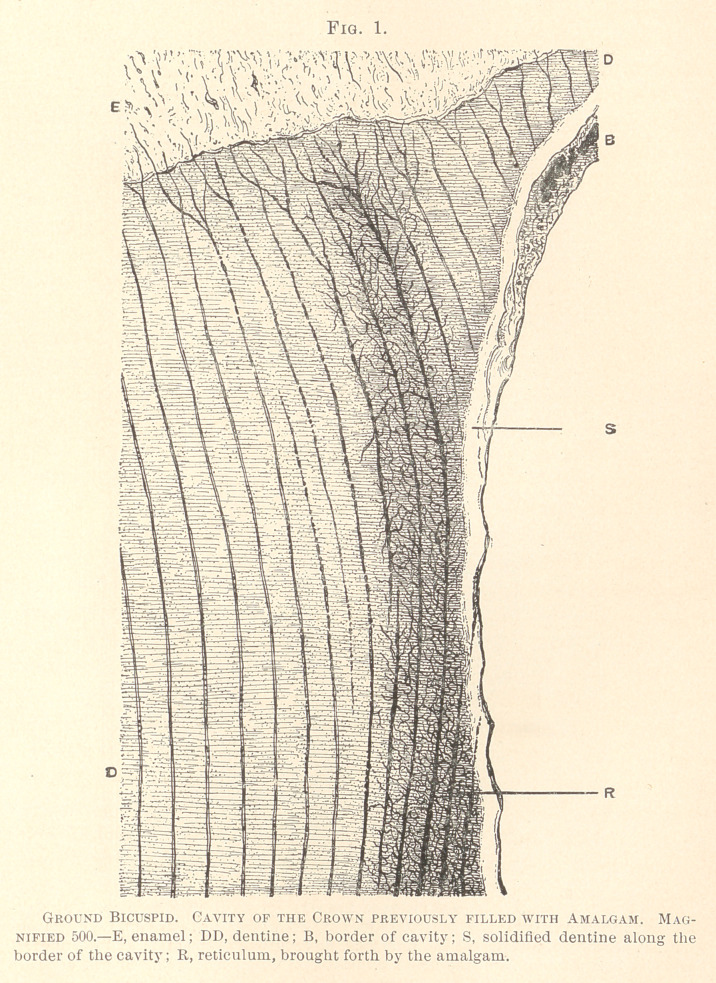


**Fig. 2. f2:**